# Effects of Elevated Root-Zone CO_2_ on Root Morphology and Nitrogen Metabolism Revealed by Physiological and Transcriptome Analysis in Oriental Melon Seedling Roots

**DOI:** 10.3390/ijms21030803

**Published:** 2020-01-25

**Authors:** Xinyu Chen, Zepeng Yin, Yang Yin, Chuanqiang Xu, Wanxin Wang, Yiling Liu, Tianlai Li

**Affiliations:** 1 College of Horticulture, Shenyang Agricultural University, Shenyang 110866, China; chenxinyuangul@163.com (X.C.); 2018500066@syau.edu.cn (Z.Y.); yinyang20200202@163.com (Y.Y.); xuchuanqiang@hotmail.com (C.X.); awan0606@163.com (W.W.); 2 Key Laboratory of Protected Horticulture Ministry of Education, Shenyang 110866, China; 3 National & Local Joint Engineering Research Center of Northern Horticultural Facilities Design & Application Technology, Shenyang 110866, China

**Keywords:** oriental melon, elevated root-zone CO_2_, root morphology, nitrogen metabolism, transcriptome

## Abstract

Rhizosphere CO_2_ is vital for crop growth, development, and productivity. However, the mechanisms of plants’ responses to root-zone CO_2_ are unclear. Oriental melons are sensitive to root-zone gas, often encountering high root-zone CO_2_ during cultivation. We investigated root growth and nitrogen metabolism in oriental melons under T1 (0.5%) and T2 (1.0%) root-zone CO_2_ concentrations using physiology and comparative transcriptome analysis. T1 and T2 increased root vigor and the nitrogen content in the short term. With increased treatment time and CO_2_ concentration, root inhibition increased, characterized by decreased root absorption, incomplete root cell structure, accelerated starch accumulation and hydrolysis, and cell aging. We identified 1280 and 1042 differentially expressed genes from T1 and T2, respectively, compared with 0.037% CO_2_-grown plants. Among them, 683 co-expressed genes are involved in stress resistance and nitrogen metabolism (enhanced phenylpropanoid biosynthesis, hormone signal transduction, glutathione metabolism, and starch and sucrose metabolism). Nitrogen metabolism gene expression, enzyme activity, and nitrogen content analyses showed that short-term elevated root-zone CO_2_ mainly regulated plant nitrogen metabolism post-transcriptionally, and directly inhibited it transcriptionally in the long term. These findings provided a basis for further investigation of nitrogen regulation by candidate genes in oriental melons under elevated root-zone CO_2_.

## 1. Introduction

For plants to grow normally, a good rhizosphere gas environment is required. The CO_2_ concentration changes continuously with different soil aeration conditions, which has a great impact on the growth, development, and yield of crops. The CO_2_ concentration in the soil close to the plant root system often reaches values up to ten-fold that of the ambient atmosphere [1,2,3]. Root and soil microorganisms produce CO_2_ through respiration, which accumulates in the root zone at concentrations normally between 0.2% and 0.5%, but can reach 20% under special circumstances [4]. The actual CO_2_ concentration in the soil also depends on the soil water content, soil type, soil depth, microbial biomass, and the activities of soil microorganisms. Responses to high CO_2_ soil environment have received increased attention recently in several crop species [5,6,7,8]. However, little information is available regarding the molecular mechanisms of plants in response to elevated root-zone CO_2_ conditions, especially at the transcriptome level.

The effects of excessive root-zone CO_2_ on plant growth, nutrient absorption, and utilization vary with plant species [9]. Nitrogen is an essential macronutrient for plant growth and basic metabolic processes. High levels of CO_2_ in the root-zone promoted the growth of tomato seedlings and increased their NO_3_^−^ uptake, especially under salinity stress and high air temperature [4,10]; however, there was no significant difference in NH_4_^+^. In lettuce, high levels of root-zone CO_2_ could alleviate the midday depression of photosynthesis and negative impacts of high air temperature on photosynthesis [11], and promoted NO_3_^−^ uptake and the growth of lettuce plants in the greenhouse [12,13]. By contrast, high root-zone soil CO_2_ had a negative impact on morphological and physiological indicators, such as plant height, root length, chlorophyll content, photosynthesis rate, stomata conductance, and NO_3_^−^ absorption and assimilation in soybean [14], maize [5], barley [15], and bean [7]. Previous studies have implied that elevated root-zone CO_2_ acted as a weak acid, causing acidification in root cells, and inhibition of nutrient uptake and the root respiration rate [16]. Moreover, a high soil CO_2_ concentration itself might be toxic to plant growth in many plant species, and under certain conditions, CO_2_ toxicity is a more important factor in plant growth than O_2_ deficiency [6]. Thus, elevated CO_2_ concentrations in the root-zone could have either positive or negative consequences for plant growth. The differences in the effects of root-zone CO_2_ on plants could be caused by differences in plant species, treatment time, the plant developmental period, and the CO_2_ concentration applied [11,17].

The oriental melon (*Cucumis melo* var. *makuwa* Makino) is one of main agricultural products that is widely cultivated in some eastern Asian countries. It is sensitive to the root-zone gas environment, and often suffers from root-zone low O_2_ and high CO_2_ stress in irrigated field cultivation. The responses of melon to root-zone hypoxia have been widely reported [18,19]. By contrast, there is little information on the mechanism of the oriental melon’s response to elevated root-zone CO_2_, especially at the transcriptome level. In addition, the molecular mechanism of the influence of root-zone CO_2_ on plant growth and mineral nutrient absorption has not been definitively proved.

The present study aimed to explore the molecular mechanism of root nitrogen metabolism in the oriental melon under elevated root-zone CO_2_. We designed an aeroponic culture system that could automatically control the root-zone CO_2_ concentration. Based on a transcriptome analysis in roots, we investigated the root morphology and root tip cell ultrastructure under different CO_2_ concentrations, including three treatments: Ambient air 0.037% (control check (CK)), elevated CO_2_ concentrations 0.5% (T1), and 1.0% (T2). Differentially expressed genes (DEGs) involved in nitrogen metabolism in different CO_2_ concentrations were screened by combining the changes of root morphology and the physiological index. Moreover, we analyzed the activities of nitrogen metabolism enzymes, and validated the sequencing accuracy of key genes using quantitative real-time PCR (qPCR). The results provided a reasonable basis to further investigate the functions of candidate genes, their transcriptional regulation, and the effective regulation of nitrogen in the oriental melon under elevated root-zone CO_2_.

## 2. Results

### 2.1. Effects of Elevated Root-Zone CO_2_ on Root Morphology

**Figure 1:**
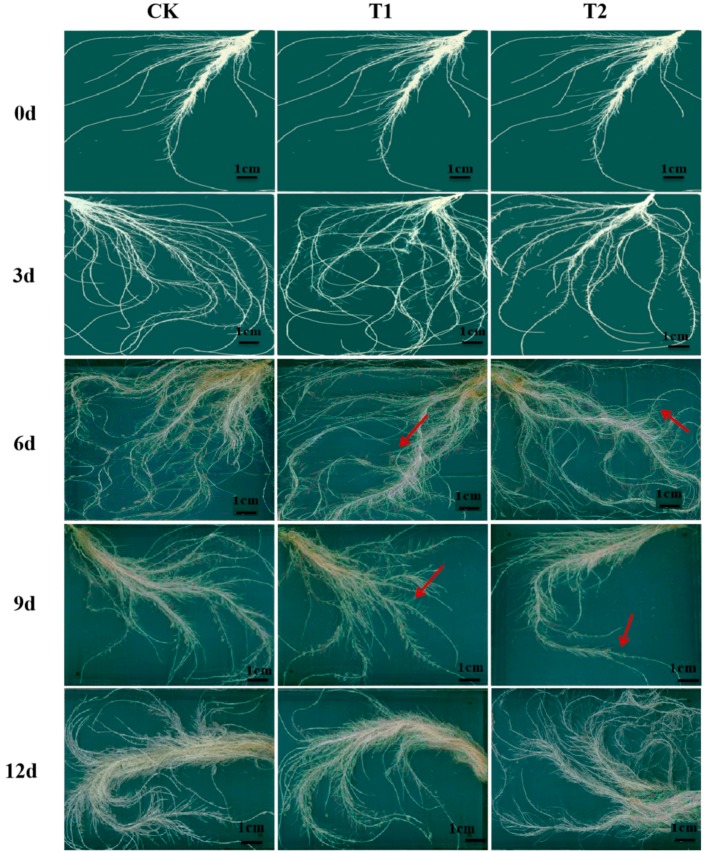
Effect of root-zone CO_2_ concentration on root morphology. Root morphology of oriental melon seedlings on day 0, 3, 6, 9, and 12 of high concentration rhizosphere CO_2_ treatment. Scale bar: 1 cm. The red arrows in the picture indicated that, the root growth under T1 and T2 treatment were not significantly different at 6 day, compared with plants under CK (control check, ambient air 0.037%); on the ninth day, the root length, root surface area, and the number of major absorbing roots under T1 and T2 treatments were remarkably lower than in the CK group.

Root morphology analysis (Figure 1; Table 1) showed that plants grown under elevated root-zone CO_2_ treatment had longer roots, a greater number of total root tips, and a larger root surface area at 3 day of treatment compared with those under ambient CO_2_ concentrations, although the resistance against elevated root-zone CO_2_ began to decline on the sixth day of treatment. The root length, the root surface area, and the number of roots with a diameter of 0.5–2.0 mm under T1 and T2 treatment were not significantly different at 6 day, compared with those of plants under CK (the red arrows indicated). On the ninth day, the root length, root surface area, and the number of major absorbing roots with diameter of 0–0.5 mm were remarkably lower under T1 and T2 treatments than in the CK group (the red arrows indicated). The main root length was reduced by 8.43% and 20.90%, respectively, and root surface area decreased by 20.35% and 52.05%, respectively, compared with the CK group. The data indicated that root growth was enhanced during short-term high CO_2_ exposure; however, with prolonged treatment, inhibitory effects were more significant.

### 2.2. Effects of Elevated Root-Zone CO_2_ on Root Vigor, Root Volume, and Plant Biomass

As shown in Table 2, the root vigor under elevated root-zone CO_2_ treatment increased initially and then decreased compared with that in the control. In the first six days of treatment, the root activity was significantly higher than that of the CK group, which indicated that elevated root-zone CO_2_ caused emergent responses of roots. With the extension of treatment time, the root responses to elevated root-zone CO_2_ decreased. The growth index (such as root volume and plant biomass) decreased significantly under T1 and T2 treatment at 9 day, compared with those under ambient levels of CO_2_. The root volumes of the T2 treatment group at 6 day, and the root volume of T1 treatment group at 9 day, were both significantly lower than those of the CK group. Root volumes of T1 and T2 at 12 day were significantly lower than those of the CK group. The shoot and root biomass accumulation of oriental melon seedlings were inhibited under high root-zone CO_2_ treatment compared with those in the CK group. At 9 day, the biomass of both the roots and the shoots were significantly inhibited by T1 and T2 treatment, and the difference increased with prolonged treatment time.

### 2.3. Effects of Elevated Root-Zone CO_2_ on Root Tip Cell Ultrastructure

**Figure 2:**
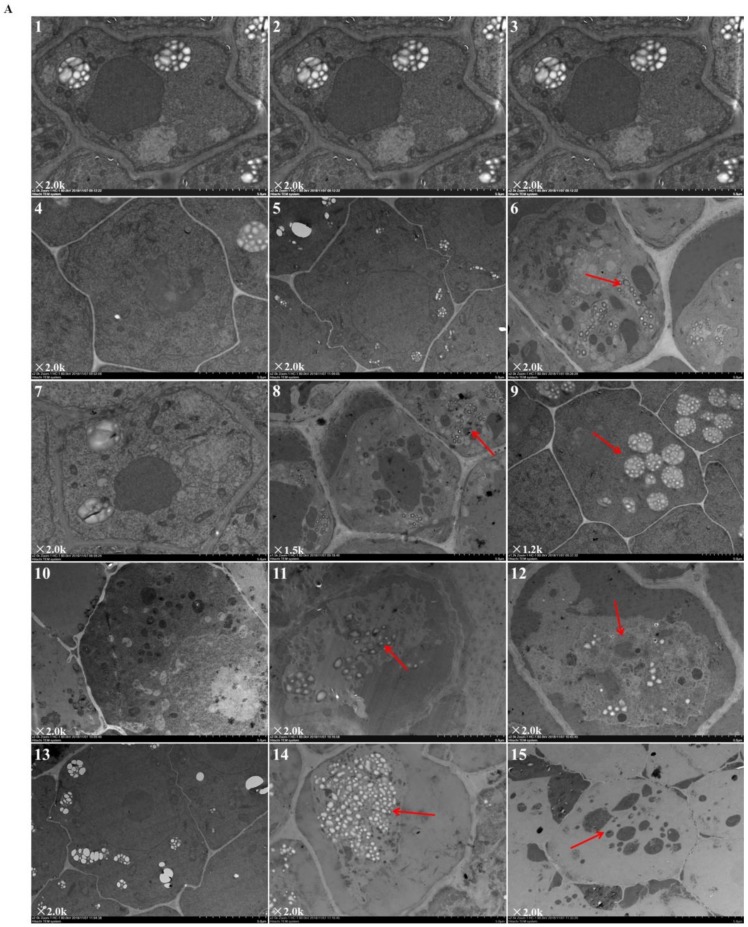

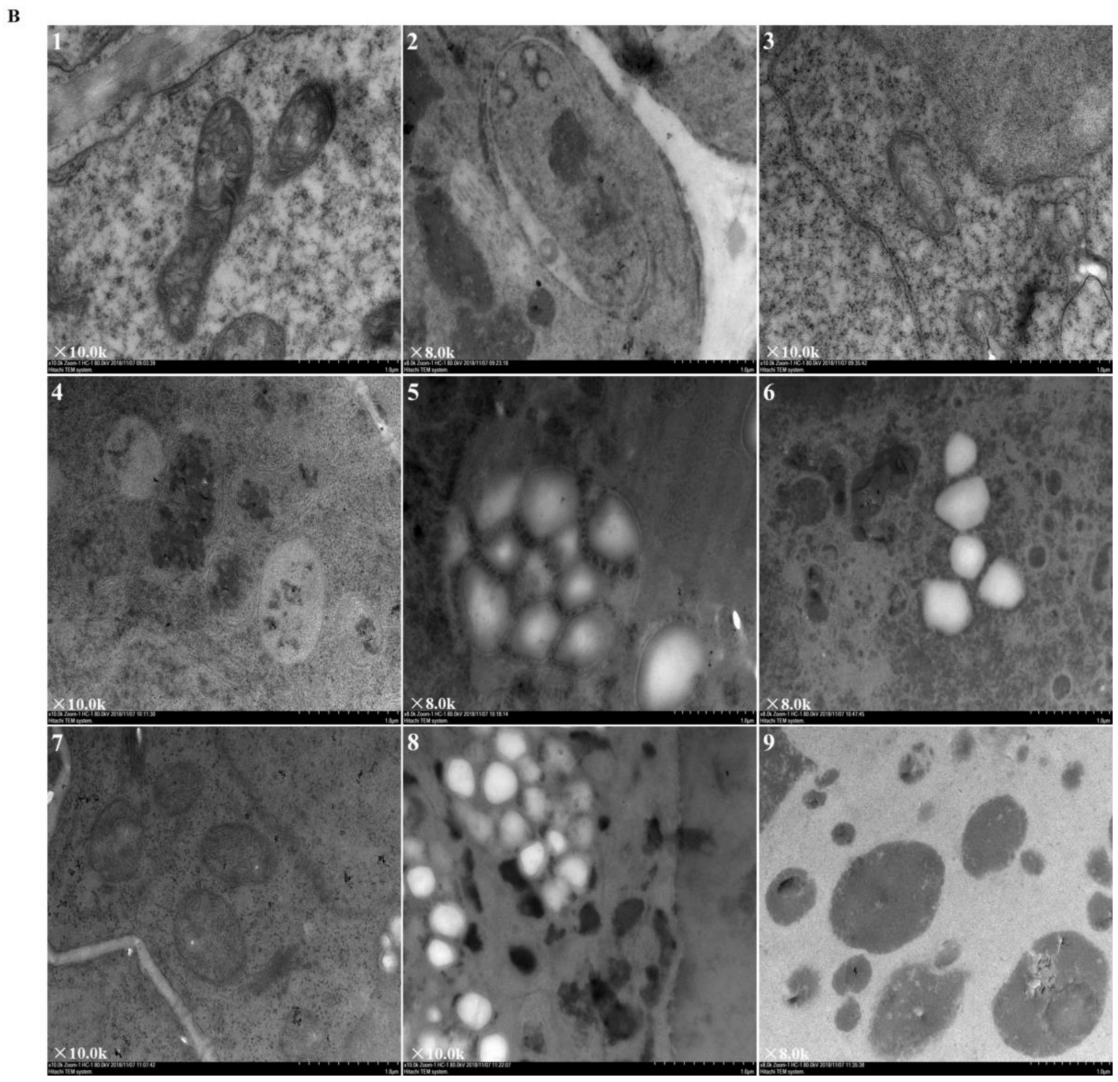
Ultrastructure of root tip cells of melon seedlings showing the effects of root-zone CO_2_ concentration. (**A**) Changes in intact root cells of oriental melon seedlings on day 0, 3, 6, 9, and 12 of high concentration rhizosphere CO_2_ treatment. There is a magnification in the lower left corner of each panel. Scale bar: 5.0 μm. The red arrow in 6 indicated that a small amount of starch grains accumulated after T2 treatment for three days; red arrows in 8, 9 indicated that at 6 day after treatment, T1 revealed fewer mitochondria and secretory vesicles emerged in plastids with very little accumulation of starch grains, T2 revealed accumulation of starch grains but a large number of organelles disappeared; red arrows in 11, 12 indicated that after 9 day of continuous treatment, T1 and T2 revealed a significant decline in the number of organelles, the accumulation of starch grains within cells increased gradually for T1, and starch grains started to be released from T2 cells; red arrows in 14, 15 indicated that after 12 day of treatment, starch grains within cells increased massively in the T1 group, together with a substantial reduction in organelles, whereas most of starch grains within T2 cells had been released, and organelles had disappeared. (**B**) Changes in organelles in root cells of oriental melon seedlings. There is a magnification in the lower left corner of each panel. Scale bar: 1.0 μm. 1, 2, 3: Changes in mitochondria on day 6 of treatment; 4: Mitochondria on day 9 of CK (ambient CO_2_) treatment; 5, 6: Starch granules produced by plastids on day 9 of T1 and T2; 7: Mitochondria on day 12 of CK; 8: Starch granules produced by plastids on day 12 of T1; 9: The plastid membrane remaining after starch hydrolysis in T2.

**Figure 3:**
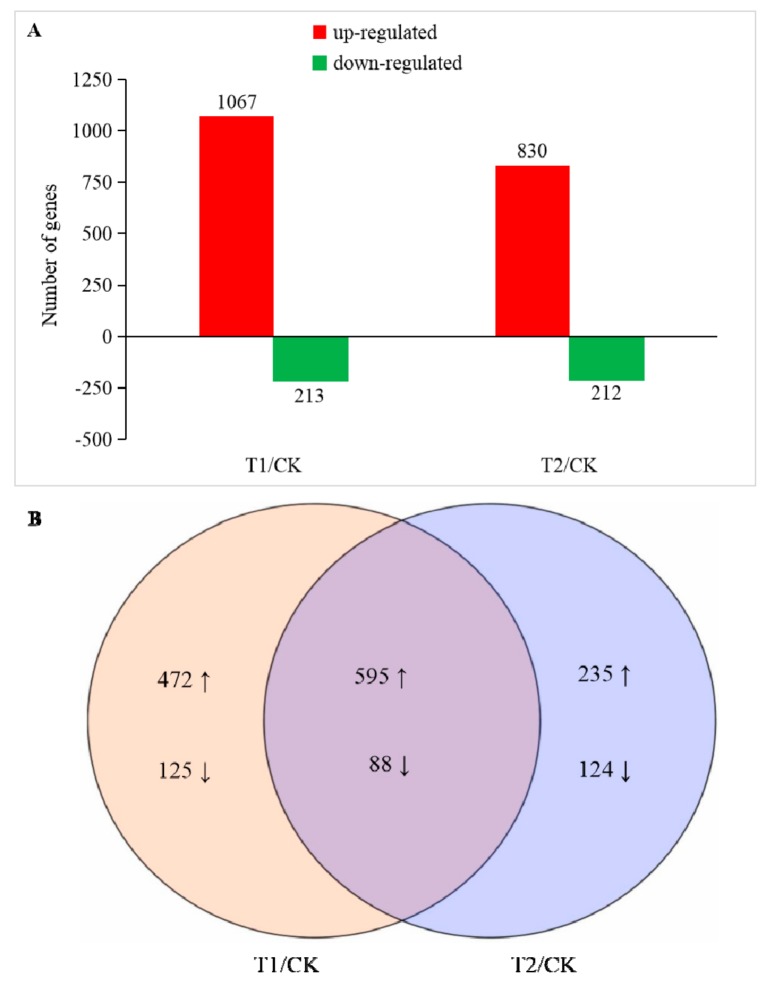
Number of differentially expressed genes (DEGs) in T1 and T2 compared with CK. (**A**) Number of up and downregulated DEGs in T1 and T2 compared with CK, respectively. (**B**) Venn-diagram of significantly different DEGs identified in T1/CK and T2/CK. The significance of the DEGs was measured using the criteria of |Log_2_FoldChange| ≥ 1 and false discovery rate (FDR) ≤ 0.05.

**Figure 4:**
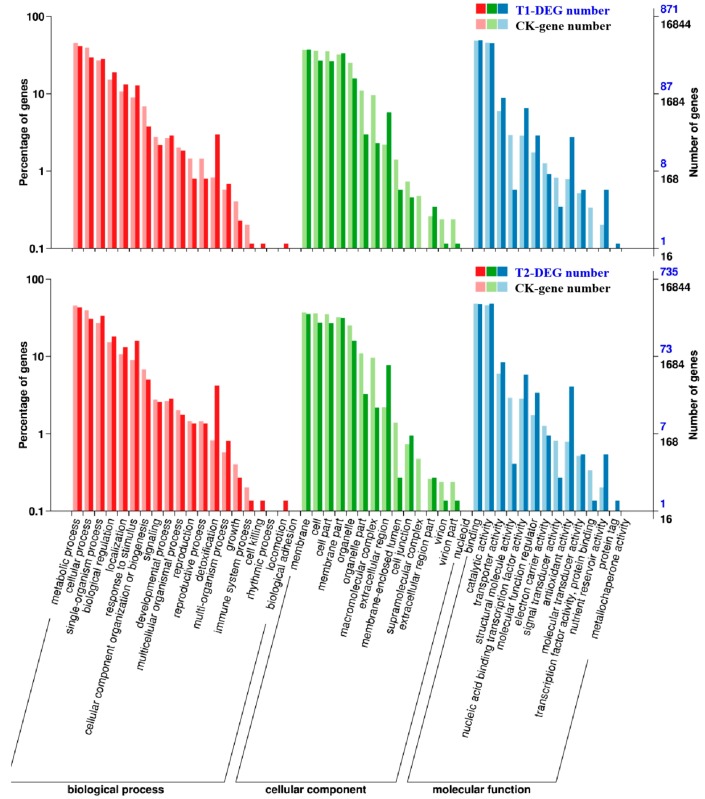
Functional classification and enrichment analysis of differentially expressed genes (DEGs) in the gene ontology (GO) analysis. Significantly enriched GO classifications were analyzed using pairwise comparisons of T1/CK and T2/CK with the criterion of false discovery rate (FDR) ≤ 0.05. The DEGs were assigned into three classifications: Biological process, cellular components, and molecular function. The *x*-axis represents GO classifications, the *y*-axis on the left represents the DEGs that account for the percentage of every classification, and the *y*-axis on the right represents the amount of DEGs in each classification.

**Figure 5:**
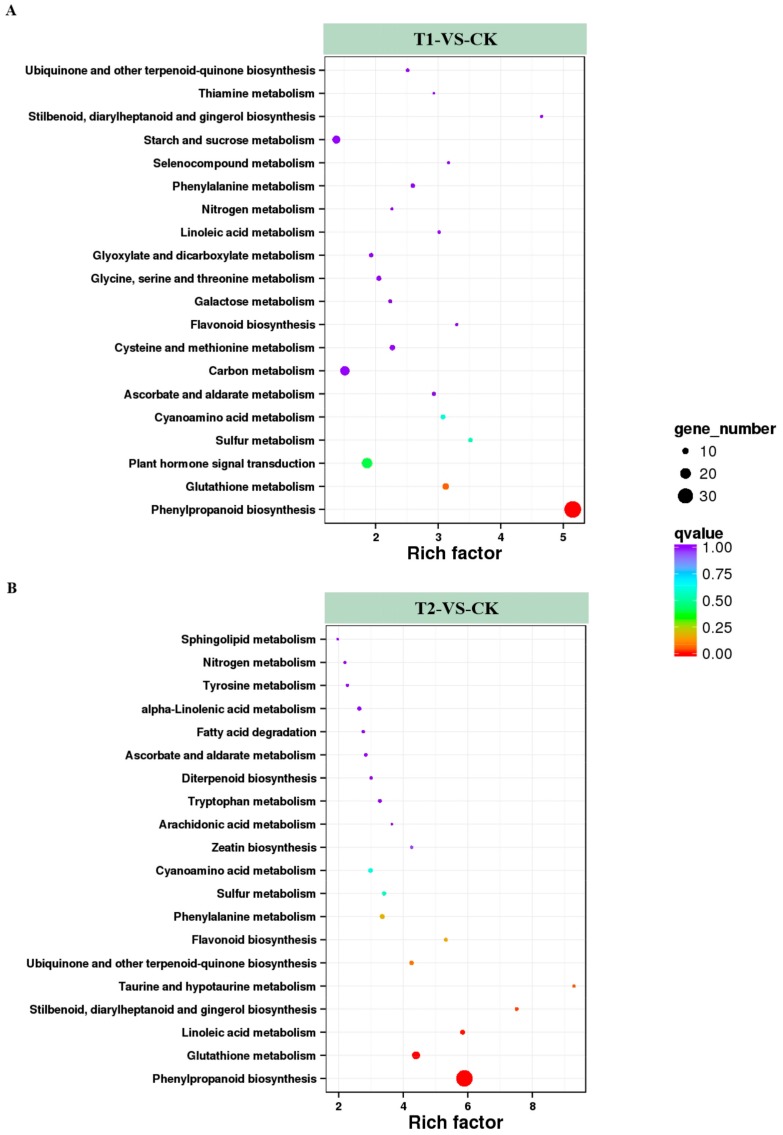
Enrichment analysis of KEGG (Kyoto Encyclopedia of Genes and Genomes) pathways. The top 20 pathways with the highest enrichment levels are shown according to the amount and enrichment level of DEGs annotated in T1 (**A**) and T2 (**B**) by pairwise comparisons to CK. The *x*-axis represents the rich factor, which was measured by the Q value and was proportional to the enrichment level. A deeper color represents the higher significance of the DEGs. The *y*-axis represents the KEGG pathways. A bigger size of spot represents more DEGs enriched.

**Figure 6:**
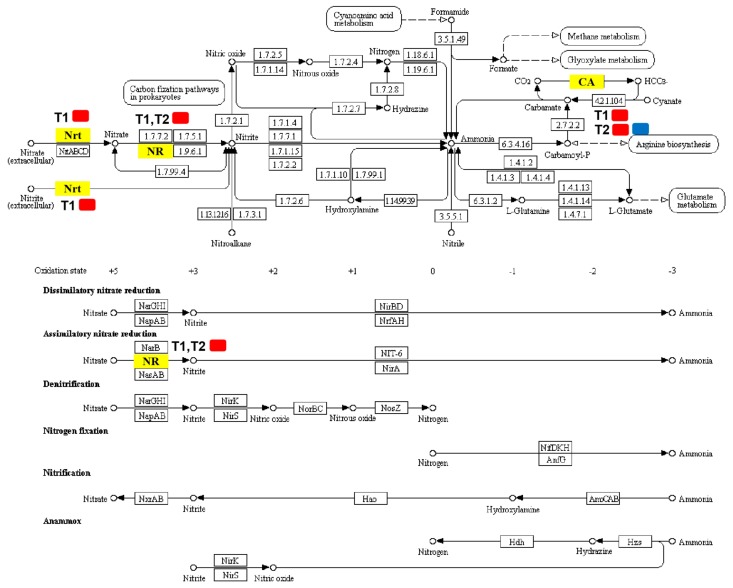
Pathways map of DEGs annotated in nitrogen metabolism. Yellow boxes represent significantly regulated DEGs annotated in T1/CK and T2/CK. Red represents a high expression level and blue represents a low expression level. Nrt (High-affinity nitrate transporter 2.2; MELO3C000140.2) was upregulated in T1; NR (Nitrate reductase; MELO3C010444.2) was upregulated in both T1 and T2; CA included alpha carbonic anhydrase (MELO3C009476.2), which was upregulated in both T1 and T2, and alpha carbonic anhydrase 7-like (MELO3C024476.2), which was downregulated only in T2.

**Figure 7:**
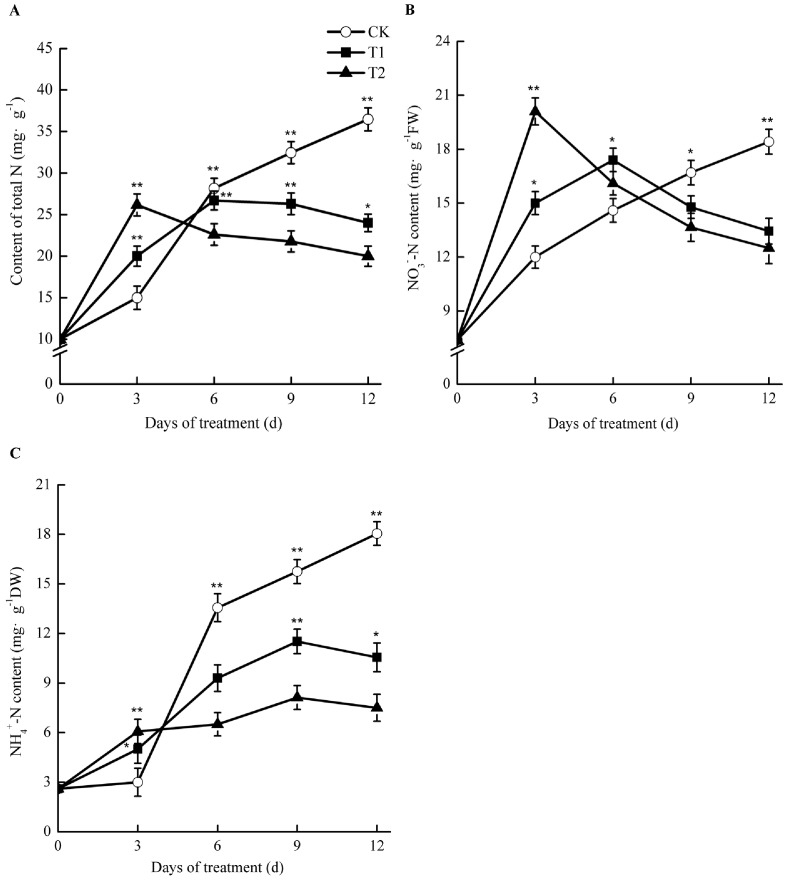
Effect of root-zone CO_2_ concentration on total N (**A**), NO_3_^−^-N (**B**), and NH_4_^+^-N (**C**) of oriental melon seeding roots. The error bars represent the standard error (SE) of the means. Significant differences between control and treatment are marked with asterisks (* represents *p* < 0.05 and ** represents *p* < 0.01).

**Figure 8:**
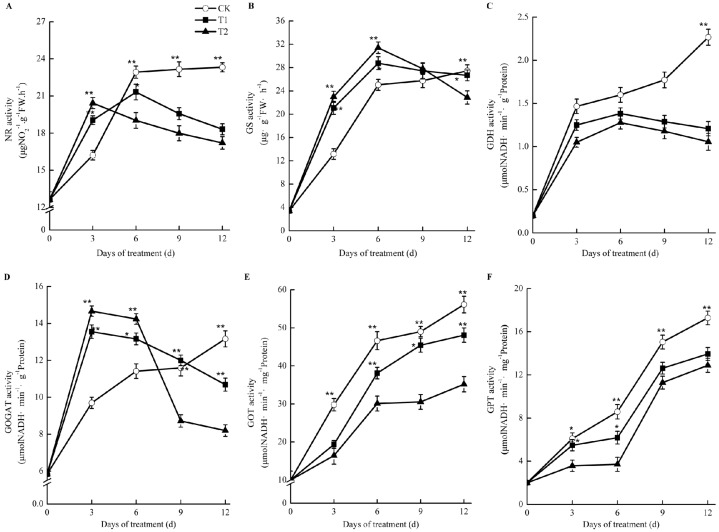
Effects of root-zone CO_2_ concentration on the activity of nitrogen metabolism enymes of oriental melon seeding roots. (**A**) nitrate reductase (NR). (**B**) glutamine synthetase (GS). (**C**) glutamate dehydrogenase (GDH). (**D**) Glutamate synthase (GOGAT). (**E**) glutamic acid oxaloacetate transa-minase (GOT) and (**F**) glutamic acid-pyruvic acid transaminase (GPT). The error bars represent standard error (SE) of the means. Significant differences between control and treatment are marked with asterisks (* represents *p* < 0.05 and ** represents *p* < 0.01).

**Figure 9:**
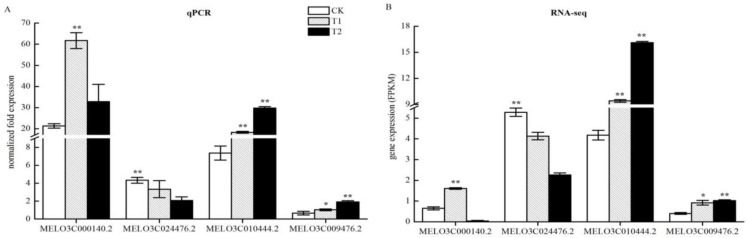
Quantitative real-time polymerase chain reaction (qPCR) analysis of differential gene expression patterns in T1 and T2 compared with CK. (**A**) Validation of sequencing accuracy of T1 and T2 by qPCR analysis in nitrogen metabolism. The relative expression levels of three DEGs identified by RNA-seq analysis are shown in (**B**). The gene expression analysis was performed based on three biological and technical replicates, respectively. “**” and “*”: Significantly different at levels of 0.01 and 0.05 by Duncan’s multiple range test, respectively. The error bars represent the standard error (SE) of the means.

Figure 2 revealed the ultrastructural changes to root tip cells under elevated root-zone CO_2_ at 0, 3, 6, 9, and 12 day after treatment. The structures of cellular organelles were intact under CK treatment (Figure 2A), with plentiful cytoplasm, nuclear envelope, and abundant cell organelles, as well as with clear organelle structure, rich mitochondrion, and distinct inner cristae. However, cellular structures under T1 and T2 showed variable changes. At 6 day after treatment (as indicated by the red arrow in Figure 2A-8,9; Figure 2B-1,2,3), compared with CK, T1 revealed fewer mitochondria with unclear structures, and secretory vesicles emerged in plastids with very little accumulation of starch grains. T2 revealed more obvious changes in cellular content, in which a large number of organelles disappeared, with no recognizable mitochondrion or Golgi apparatus structure, but more accumulation of starch grains. After 9 day of continuous treatment, compared with those in the CK group, T1 and T2 revealed a significant decline in the number of organelles (as indicated by the red arrow in Figure 2A-11,12; Figure 2B-4,5,6). Meanwhile, the accumulation of starch grains within cells increased gradually for T1, and starch grains started to be released from T2 cells. Then, after 12 day of treatment (as indicated by the red arrow in Figure 2A-14,15; Figure 2B-7,8,9), starch grains within cells increased massively in the T1 group, together with a substantial reduction in organelles, such as mitochondria and Golgi apparatus, whereas most of the starch grains within T2 cells had been released, and organelles had disappeared. The results indicated that elevated root-zone CO_2_ contributed to starch grain accumulation and mitochondrion structural changes. Higher CO_2_ concentrations and longer treatment time caused more obvious changes, with starch grain accumulation and more rapid hydrolysis within cells.

### 2.4. Identification of Differentially Expressed Genes (DEGs)

According to the microscopic observations and physiological index analysis above, root tips at nine days after treatment were sampled for RNA-sequencing (RNA-seq) analysis. Through transcriptome sequencing, we obtained a total of 441.23 million raw sequencing reads, which were filtered after mass analysis. We finally obtained 220.61 million clean paired end reads. Further analysis showed that 93.21% to 94.54% of the clean reads were located in the reference genome at a standard deviation of 0.01. Using the Gffcompare program in String Tie, 22,462 genes were identified [20] with a quality of > 30 (Q30), thus ensuring the quality and reliability of the sequencing data obtained.

The expression level of the obtained sequencing data was analyzed using DEseq, and a gene meeting the condition of FDR (false discovery rate) < 0.05 and |Log_2_FoldChange| ≥ 1 was defined as a significant DEG. The gene libraries obtained in T1 and T2 were compared with CK, and two DEG libraries were obtained as T1/CK and T2/CK. A total of 1639 DEGs were identified in the two libraries (Table S1). In T1 and T2, 1302 genes were upregulated, and 337 genes were downregulated (Figure 3). In T1, we found a significant difference in the expression levels of 1280 genes, of which 1067 were upregulated and 213 were downregulated. The number of DEGs gene in T2 was reduced compared with that in T1. There were 1042 DEGs, of which 830 were upregulated and 212 were downregulated. In addition, there were 597 genes that only showed significant differences in their expression levels in T1, while there were only 359 genes that only showed significant differences in their expression levels in T2. From the sequencing results, the expression of a large number of genes in the root cells of melon seedlings were changed by different degrees under the influence of a high concentration of rhizosphere CO_2_. Changes in the expression of these genes could change a series of metabolic processes in the cells at the molecular level, thereby affecting the physiology of the entire plant.

### 2.5. Functional Classification and Enrichment Analysis on Gene Ontology (GO)

Gene ontology (GO) was used for functional classification and enrichment analysis of the DEGs as identified above. In T1 and T2, 871 and 735 differentially expressed genes were annotated to various GO functions. Then, their annotated gene functions were divided into three categories (Figure 4), “biological processes”, “cell components”, and “molecular function”. Some genes had more than one functional term in the GO functional classification. The results of the analysis indicated that most of the identified DEGs were distributed in the “molecular function” category of GO, in which the number of DEGs involved in processes such as “binding”, “catalytic activity”, “organic cyclic compound binding”, and “ion binding” accounted for a large percentage in both T1 and T2, the highest numbers of DEGs were found in “binding” and “catalytic activity”. Therefore, we speculated that high concentrations of CO_2_ might have greater impacts on protein synthesis and functional modification processes. In addition, in the two treatments, the “biological processes” had the most DEGs in the “metabolic process”, which contained genes related to nitrogen metabolism and abiotic stress resistance in plants, indicating that the stress probably affected the overall metabolic process of melon seedlings and stimulated their self-defense functions. In the “cellular component” section, the most enriched functions were “cellular process” and “membrane”, which mainly covered genes related to the plasma membrane and organelle synthesis. Therefore, we speculated that it had something to do with the accelerated aging of root cells and the functionally restricted plasma membrane under CO_2_ stress. Comparing T2 with T1, the DEGs of the “immune system process”, “cell junction”, “extracellular region part”, “nucleoid”, and “structural molecule activity” were decreased. These genes are closely related to the resistance of plants, such that we speculated that the excessive root-zone CO_2_ might reduce the root resistance of melon seedlings, which might explain why the plant roots were weaker in T2.

### 2.6. KEGG (Kyoto Encyclopedia of Genes and Genomes) Analysis of DEGs

The DEGs were loaded onto the KEGG platform to further identify the corresponding metabolic pathways, and the enrichment levels of DEGs in different metabolic pathways were also counted. Among the DEGs in T1 and T2, 364 and 333 genes, respectively, were annotated into different KEGG pathways. Among these annotated genes, DEGs in T1 were involved in 84 KEGG metabolic pathways, and the genes in T2 covered 73 metabolic pathways. Among these metabolic pathways, 20 KEGG pathways containing the highest number of DEGs and the highest enrichment levels were selected and listed in Figure 5. Among them, pathways related to nitrogen metabolism, such as nitrogen metabolism (ko00910) and cyanoamino acid metabolism (ko00460), were identified in both T1/CK and T2/CK. Others such as ascorbate and aldarate metabolism (ko00053), flavonoid biosynthesis (ko00941) were also active in the two treatment conditions. These results indicated that plants might resist high CO_2_ stress by adjusting stress resistance, and antioxidant and signal transduction processes. In addition, high CO_2_ stress had significant effects on the metabolism of nitrogen, sulfur, and some amino acids. Next, the five KEGG pathways with the highest degree of DEG enrichment in the two CO_2_ concentration treatments were analyzed. Four pathways were found in both treatments, namely “phenylpropanoid biosynthesis”, “plant hormone signal transduction”, “carbon metabolism”, and “starch and sucrose metabolism”. The synthesis of phenylpropanoids and the transduction of hormonal signals are related to the resistance of plants. At the same time, the changes in genes involved in carbon metabolism, starch, and sucrose metabolism might be the main reasons for the accelerated hydrolysis of starch in root cells under high CO_2_ stress. In addition, the rich factor of the DEGs in T1 was 5.15, and in T2 it was up to 9.29. The metabolic pathways with higher rich factor included “phenylpropanoid biosynthesis” and “stilbenoid, diarylheptanoid and gingerol biosynthesis”. It demonstrated that melon seedlings might resist root-zone CO_2_ stress via metabolism of the above compounds. At the same time, we found that the enrichment level of the nitrogen metabolism pathway was higher in both T1 and T2, with rich factors of 2.26 and 2.19, respectively, indicating that the increase of CO_2_ in root zone had significant effects on the nitrogen metabolism of melon seedling roots.

### 2.7. DEGs Involved in Nitrogen Metabolism

Differentially expressed genes associated with nitrogen metabolism were screened out (Table 3; Figure 6), which identified four DEGs in T1 and T2. For T1, there were three DEGs and all of them were upregulated: High-affinity nitrate transporter 2.2 (MELO3C000140.2), nitrate reductase (MELO3C010444.2), and alpha carbonic anhydrase (MELO3C009476.2). For T2, except for alpha carbonic anhydrase 7-like (MELO3C024476.2), which was downregulated considerably, the expression levels of the remaining two nitrogen metabolism-related DEGs increased significantly, including nitrate reductase (MELO3C010444.2) and alpha carbonic anhydrase (MELO3C009476.2).

### 2.8. Effects of Elevated Root-Zone CO_2_ on Nitrogen Concentration

Under elevated root-zone CO_2_, the contents of total nitrogen, nitrate nitrogen (NO_3_^−^-N), and ammonium nitrogen (NH_4_^+^-N) in the roots of oriental melons all showed a trend of an early increase and a later reduction (Figure 7). After 3 day of treatment, the concentrations of NO_3_^−^-N, NH_4_^+^-N, and total nitrogen in roots of T1 and T2 were significantly higher than those of CK. For T1 and T2, the NH_4_^+^-N content experienced the greatest increase, at 1.67 and 2.02 times as much as that of CK, respectively. For T1, the total nitrogen and NO_3_^−^-N contents were 33.33% and 25.00% higher than those of CK, respectively, while for T2, they were 41.13% and 67.50% higher than those of CK, respectively (*p* < 0.05). As the treatment time increased, nitrogen accumulation in T1 and T2 started to decline. After 6 day of treatment, NH_4_^+^-N and total nitrogen in the roots under elevated root-zone CO_2_ were lower than those of CK, although NO_3_^−^-N was still higher than that of CK, and began to decline after 6 day. After 9 days of treatment, total nitrogen, NO_3_^−^N, and NH_4_^+^-N levels were all lower than those of CK at 18.95%, 11.5%, and 26.86% respectively, in T1 compared with those in CK, while they decreased by 32.91%, 18.26%, and 48.44%, respectively, in T2 compared with those in CK.

Such results suggested that elevated root-zone CO_2_ at the early stage facilitated NO_3_^−^-N uptake for melon roots, causing a temporary accumulation of NH_4_^+^-N in the roots. However, as the treatment time increased, the NO_3_^−^-N uptake capacity of roots declined, and nitrogen accumulation in roots declined accordingly. The higher the treatment concentration applied, the greater the difference observed.

### 2.9. Effects of Elevated Root-Zone CO_2_ on Enzyme Activities Related to Nitrogen Metabolism

As shown in Figure 8, the activities of nitrate reductase (NR), glutamine synthetase (GS), and glutamate synthase (GOGAT) increased at first and then decreased compared with those in the control in oriental melon seedling roots during treatment with elevated root-zone CO_2_. At the thrid day of treatment, the NR activity under elevated root-zone CO_2_ treatment was significantly higher than that of the control. With prolonged treatment, the activity of NR under elevated root-zone CO_2_ treatment decreased gradually. The NR activities of T1 and T2 treatment were significantly lower than those of the control at the sixth day of treatment at 7% lower than the control under T1 treatment and 17% lower than the control under T2 treatment (Figure 8A). GS and GOGAT activities were significantly higher than the control at six days after treatment, and then began to decrease. The higher the CO_2_ concentration, the earlier the inhibition appeared. After nine days of treatment, the GOGAT activity under T2 treatment was 25% lower than that of the control, while the GOGAT activity under T1 treatment was still slightly higher than that of the control. After twelve days of treatment, the activities of GS and GOGAT in the roots treated under T1 and T2 were lower than those of the control (Figure 8B,D). The activities of glutamate dehydrogenase (GDH) (Figure 8C), glutamic acid oxaloacetate transaminase (GOT) (Figure 8E), and glutamic acid-pyruvic acid transaminase (GPT) (Figure 8F) in oriental melon seedling roots were significantly lower than those of the control under elevated root-zone CO_2_ treatment.

### 2.10. QPCR Verification of the Accuracy of Changes in Expression of DEGs Related to Nitrogen Metabolism

Transcriptome sequencing identified nitrogen metabolism-related DEGs in the roots of melon seedlings under high CO_2_ stress. To test whether the RNA-seq results were reliable, we verified the four genes using quantitative qPCR technology. The resulting gene expression results were consistent with those shown by RNA-seq (Figure 9), with few differences. This result showed that the RNA-seq data were reliable and could be used for further analytical studies.

## 3. Discussion

### 3.1. Elevated Root-Zone CO_2_ Affected the Root Growth and Morphological Structure of Melon Seedlings

Plant root morphological configuration is an important factor that allows plants to obtain nutrients, and root morphogenesis is influenced by genetic factors and the growth environment [21,22]. The root tip is an important part of the root system in terms of water and nutrient absorption. The higher the number of root tips, the longer the root length, and the larger the root surface area, the more beneficial it is to the effective absorption of root nutrients [22,23]. The present study showed that an increase of the rhizosphere CO_2_ concentration in the oriental melon had a certain promoting effect on root growth in the short term (Table 1 and Table 2). The main root length, number of root tips, and root vigor were all higher than those of the control at six days after treatment. Similar results were found in earlier studies [24]. The plant roots were able to transport CO_2_ such as HCO_3_^−^ (dissolved inorganic carbon, DIC) through xylem vessels to the shoot where it could be utilized by photosynthetic tissues for the assimilation of carbon [25]. The elevated root-zone CO_2_ concentration increased the light-saturated net photosynthetic rate and photosynthetic products, which alleviated water loss and facilitated a higher turnover of Calvin cycle enzymes in the short term, allowing a greater amount of photoassimilates to be apportioned to the roots for better growth of lettuce [24]. Moreover, root morphology exhibits high plasticity, and the root system could improve the absorption rate of water and nutrients by increasing the number of root tips, or promoting the elongation of the taproot when the external environment changed. This would alleviate the direct damage to the plant caused by environmental adversity [26].

Root and shoot growth were significantly inhibited after nine days of treatment under 0.5% CO_2_ concentration or after six days under 1% CO_2_ concentration treatment (Table 1 and Table 2). Previous studies reported that plant height, root length, leaf number, leaf area, and plant biomass were reduced after the soil was exposed to high CO_2_ concentrations, compared with those in the ambient CO_2_ concentration control [5]. The plant membrane has high permeability for CO_2_. Exposure to high CO_2_ could cause acidification in the root cells, because CO_2_ is a weak acid, and plant cells rapidly take up many weak acids in the undissociated form [27,28]. As the weak acids dissociate in the cell, the cytoplasm becomes acidified, and the pH in the root system changes drastically, which would destroy the function of the root cell membrane, and the root cells would lose their ability for osmotic balance. Damaged root cells would not absorb water sufficiently, resulting in the weakening or loss of root cell function, and these impaired abilities would hinder the root system’s absorption, secretion, and synthesis functions [29]. Transcriptome analysis suggested that the metabolic pathways of phenylpropanoid biosynthesis, plant hormone signal transduction, and glutathione metabolism in melon roots were upregulated under treatment with high rhizosphere CO_2_. These results indicated that the melon root system was subjected to stress after nine days of treatment. The root system could protect itself by synthesizing substances such as phenylpropane and glutathione, and by regulating its metabolism through the transmission of hormone signals. As previously reported, when plant roots suffered CO_2_ stress, the roots produced abscisic acid and sent a hormone signal to the leaves to close stomata and prevent water loss [8].

Observation of the ultrastructure of root tip cells of melon seedlings showed that the number of organelles and inclusions in the root tip cells were reduced, the cell membrane was damaged, and the accumulation and dissolution rate of starch granules in the cells were accelerated under conditions of high rhizosphere CO_2_ (Figure 2). Transcriptome analysis also showed that the expression of genes involved in the membrane part and organelle part pathways in root cells were significantly downregulated, and the expression of genes participating in starch and sucrose metabolism were upregulated (Figure 4 and Figure 5). These results indicated that the high root-zone CO_2_ treatment damaged the organelle and membrane structure of root tip cells and decreased their normal metabolic function. Previous studies found that as a compensatory mechanism under elevated root-zone CO_2_ treatment, to increase the absorption of water, plants could hydrolyze the starch in roots and stems into soluble sugars, thereby reducing the osmotic potential to facilitate the absorption of water [30,31]. In the present study, the accelerated starch granule dissolution of root cells could be associated with this compensation response to high root-zone CO_2_ stress.

### 3.2. Elevated Root-Zone CO_2_ Affected the Accumulation and Metabolism of Nitrogen in Melon Seedlings Roots

As an important nutrient element and signaling molecule in plants, nitrogen affects many processes in plant metabolism and development [32,33]. Nitrate nitrogen is the main inorganic nitrogen form absorbed by plant roots, especially melon roots. The uptake and utilization of nitrogen by the root system affects root morphology [34]. The nitrogen metabolism process of plant roots under normal conditions comprises nitrate (NO_3_^−^) being reduced by nitrate reductase (NR) to nitrite (NO_2_^−^) after being absorbed by root cells, then under the action of nitrite reductase (NiR), ammonium (NH_4_^+^) is synthesized. NH_4_^+^ participates in the synthesis of amino acids in plants by the action of glutamine synthetase-glutamine-2-oxoglutarate aminotransferase (GS-GOGAT) [35].

Our results revealed that the contents of nitrate nitrogen, ammonium nitrogen, and total nitrogen in the root system of melon seedlings showed a trend of first increasing and then decreasing under treatment by elevated root-zone CO_2_, as compared with that of the control (Figure 7). Elevated root-zone CO_2_ had a particularly significant influence on the NO_3_^−^ concentration in the root. Previous studies indicated that a supply of enriched CO_2_ to the rhizosphere of tomato plants influenced NO_3_^−^ uptake through the formation of increased amounts of HCO_3_^−^ in the cytosol [9]. The effects of elevated CO_2_ concentration in the root-zone on nitrate absorption and metabolism are related to the membrane exchange between HCO_3_^−^ and NO_3_^−^. When the transformation of CO_2_ into HCO_3_^−^ in cytoplasm increases, the concentration gradient of HCO_3_^−^ inside and outside the plasma membrane increases, which provides the impetus for the outflow of HCO_3_^−^. The outflow of HCO_3_^−^ is synchronous with the inflow of NO_3_^−^ in the medium; therefore, the outflow of HCO_3_^−^ promotes the absorption of NO_3_^−^ [36]. The formation of intracellular HCO_3_^−^ is closely related to the activities of carbonic anhydrase (CA) [37]. Through transcriptome analysis (Table 3; Figure 6), we found that the expression of the CA MELO3C009476.2 in the melon root system was upregulated under 0.5% and 1% root-zone CO_2_. Therefore, under elevated root-zone CO_2_ concentration, the increased expression of the CA gene in the melon root promoted the synthesis of CA, resulting in an increased HCO_3_^−^ concentration in the cytoplasm, and an increase in the nitrogen content in the root system. The CA gene MELO3C024476.2 was downregulated under 1% CO_2_ treatment, which might be related to excessive stress. The increase in NO_3_^−^ further induced elevated NR activity, because NR is an inducible enzyme (Figure 8A), leading to increased NO_3_^−^ reduction to NH_4_^+^, which was responsible for the temporary accumulation of NH_4_^+^ concentration in the root system (Figure 7C).

With extended treatment time, every 10 kPa CO_2_ increase in the root cytoplasm resulted in 75–90 mmol·L^−1^ HCO_3_^−^ production. When HCO_3_^−^ in the cytoplasm reached 10 mmol·L^−1^, the activities of succinic acid dehydrogenase and cytochrome oxidase were inhibited, and the functions of root absorption, secretion, and synthesis were reduced [38]. Therefore, this resulted in a decrease in the NO_3_^−^-N content in the root system, reduced NR activity, and inhibition of the reduction of NO_3_^−^ to NH_4_^+^, finally reducing the nitrogen content in the root system. In this study, the activities of NR, GS, and GOGAT in the root system of melon seedlings under elevated root-zone CO_2_ increased at first and then decreased compared with those of the control (Figure 8). NR is an inducible enzyme that is involved in the first step in the NO_3_^−^ reduction process; therefore, in the early stage of treatment, the root system increased NO_3_^−^ absorption, induced NR activity, and promoted the reduction of NO_3_^−^ to NH_4_^+^. The coupled GS-GOGAT reaction cycle is the main channel of nitrogen metabolism [35]. The transient accumulation of NH_4_^+^ activated GS, and catalyzed the synthesis of glutamine, which in turn promoted the activity of GOGAT. The carbon source of this process might come from the rhizosphere CO_2_ [39]. With increased treatment time, the root activity decreased (Table 2), the accumulation of NO_3_^−^ and NH_4_^+^ in the root decreased, and the activities of NR, GS, and GOGAT also decreased (Figure 8).

Glutamate dehydrogenase (GDH), glutamic-oxaloacetic transaminase (GOT), and glutamic-pyruvic transaminase (GPT) are the key enzymes for plant ammonia assimilation and the formation of amino acids. Under elevated root-zone CO_2_ concentrations, the activities of GDH, GOT and GPT in melon roots were significantly lower than those in the control. In summary, an increased root-zone CO_2_ concentration inhibited the initiation of ammonia assimilation, as well as nitrogen transport, in melon roots. The synthesis of amino acids and their exchange were also restrained.

We investigated four DEGs related to enzymatic reactions of nitrogen metabolism (Table 3; Figure 6) nine days after treatment. The expression results of qPCR obtained were generally consistent with the RNA-seq results (Figure 9). MELO3C000140.2 encodes a high-affinity nitrate transporter 2.2 (NRT) and was upregulated in T1. MELO3C010444.2 encodes nitrate reductase (NR) and was upregulated in T1 and T2. However, these results were inconsistent with the enzyme activity results (Figure 8). On one hand, gene expression is regulated by many transcriptional regulators. Upregulated expression of NRT and NR were likely to be affected by the signal from aboveground glutaredoxin (CEPD1/2). When root nitrogen uptake is limited, the synthesis of C-terminally encoded peptides (CEPs) is induced and transported to the shoot. The CEPR (CEP receptor) in the shoot is activated, triggering the expression of two glutaredoxin genes: *CEP* DOWNSTREAM 1 (CEPD1) and CEP DOWNSTREAM 2 (CEPD2). CEPD1/2 act as a signal traveling back to the root to enhance NRT gene expression. Meanwhile, external NO_3_^−^ could also induce the expression of genes related to NO_3_^−^ absorption and transformation in the root system. On the other hand, protein synthesis is influenced by many factors. In plants, 14-3-3 proteins bind to and regulate enzymes involved in basic metabolic processes, such as nitrogen and carbon assimilation [40]. Degradation of 14-3-3 protein requires Ca^2+^ involvement, which is dependent on CBL-interacting protein kinase 14 (CIPK14)-mediated phosphorylation of ATL31 [41]. Our study found that MELO3C011421.2 and MELO3C011028.2, encoding 14-3-3 proteins, were down and upregulated in 0.5% and 1.0% CO_2_, respectively, and acted as a signal traveling back to roots to enhance NRT gene expression, which might have affected NR activity. Such a case indicated that the expression of these genes might be affected at the translation and post-translation modification stages, resulting in their inability to successfully synthesize proteins. It should be noted that, in general, changes in the amount of gene expression at the transcriptional stage are not always consistent with changes in the corresponding protein levels, which has been detected in many studies [42,43,44]. Gene expression is affected by a variety of factors. The final synthesis of proteins is not only regulated by transcription levels, but also by translational and post-translational regulation. The degradation of RNA caused by the environment and the selective translation of mRNAs, means that RNA obtained by transcription was not necessarily used to synthesize the corresponding protein. Meanwhile, proteins would also be inactivated or functionally altered by degradation, modification, and folding. For example, microRNAs (miRNAs) could inhibit protein expression or directly degrade mRNAs. Under stress during the plant seedling stage, post-transcriptional regulation is more likely to cause an imbalance between transcriptional expression and protein expression [45]. However, these results require further verification.

## 4. Materials and Methods

### 4.1. Plant Material and Growth Conditions

**Figure 10:**
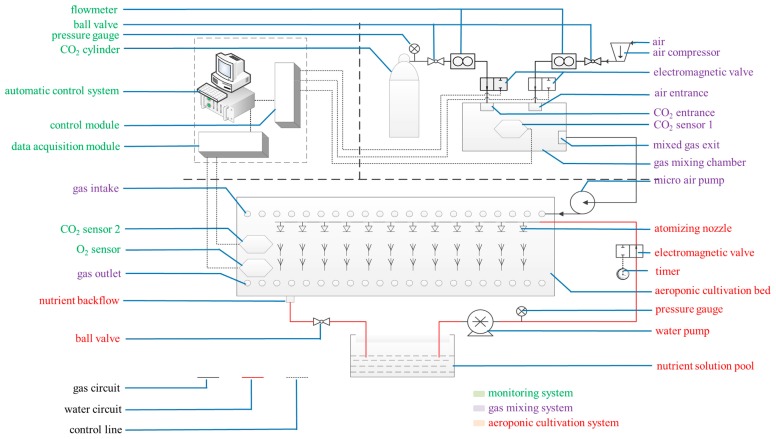
Cultivation and treatment equipment. The system mainly comprised a CO_2_-air mixing system (purple notes), a CO_2_ concentration monitoring system (green notes), and an aeroponic cultivation system (red notes).

Oriental melon cultivar “Yumeiren” (Hengyuan Seed Industry, Changchun, Jilin, China) was grown aeroponically in a solar greenhouse in the scientific research base of Shenyang agricultural university, Shenyang, Liaoning, China. The aeroponics culture system is depicted in Figure 10, with spacing of 40 cm in the rows and lines, respectively. Each bin accommodated six plants; whose basal parts of the stem were set in polyurethane cubes with sides of 15 mm. The Yamazaki solution for melon at 1/2 strength was supplied through a pump at a frequency of one spray for 50 s per 180 s during the seedling stage. The flux of the atomizing nozzle was 1 L·min^−1^ and the pipe pressure was 0.2 Pa. The solution was recycled and renewed every week. The solution temperature ranged from 21–25 °C. All oriental melon seedling shoots were cultivated vertically, and other managements were the same as the routine production management.

### 4.2. Root-Zone CO_2_ Concentration Treatments

The root-zone CO_2_ concentration treatment system mainly comprised a CO_2_-air mixing system, a CO_2_ concentration monitoring system, and an aeroponic cultivation system (Figure 10). Three weeks after transplantation, three different root-zone CO_2_ concentrations (ambient air (0.037%), and elevated CO_2_ concentrations of 0.5% and 1.0%) were supplied to separate bins. Continuous elevated CO_2_ treatment lasted for 12 days. Fresh air from the greenhouse outside was continuously circulated through the bin with the average ambient CO_2_ concentration of 0.0370% (0.037% ± 0.0005%). The elevated CO_2_ concentrations of 0.5% and 1.0% were controlled using premixed CO_2_-air mixtures from compressed air and high purity CO_2_ cylinders. The air enriched with 0.5% and 1.0% CO_2_ was supplied at 0.5 L·min^−1^ from a gas mixing chamber with separate vents to different aeroponic bins at 0.5 L·min^−1^ in the greenhouse. Elevated root-zone CO_2_ levels of 0.5%, and 1.0% were maintained using CO_2_-air mixtures. There were three bins for each root-zone CO_2_ concentration treatment. The CO_2_ sensor 1 was installed in the gas mixing chamber to monitor the mixed gas CO_2_ concentration. The computer control system regulated the intake of CO_2_ and air by analyzing the variation range of CO_2_ sensor 1, and maintained the CO_2_ concentration at the pre-set CO_2_ level in the gas mixing chamber. The fixed elevated CO_2_ level varied by about ± 0.005% from the pre-set CO_2_ level. All experiments were performed in a greenhouse kept at the same temperature. The gas was introduced through a pipe inserted near the root, with its open-end air inlet located at the bottom of the ventilation pipeline. The gas mixture was forced upwards towards the roots. The root-zone CO_2_ concentration of the seedlings could be monitored by CO_2_ sensor 2 and an oxygen sensor located in the cultivation bed. The computer data acquisition module collected the data from CO_2_ sensor 2 and the oxygen sensor in the cultivation bed, and then transmitted the data to the computer control module to ensure the CO_2_ concentration in the cultivation bed was at the pre-set CO_2_ level. The CO_2_ concentration at the shoot base was continuously monitored using an infrared CO_2_ gas analyzer (Analytical Development Corporation, Hoddesdon, UK). No significant change was found in atmospheric CO_2_ concentration, which indicated that the bin lids were airtight. The computer simultaneously carried on the real-time monitoring, data recording, and storage.

### 4.3. Measurements of the Plant Growth Index

Whole plants were divided into shoot and roots, and dried to a constant mass in an oven at 60 °C for three days to obtain the dry weight. The sampling time for all physiological indicators was 0, 3, 6, 9, and 12 days after treatment. The root volume was generally determined using the drainage method. Triphenyltetrazolium chloride (TTC) was used to measure root vigor according to Knievel’s method [46]. The values were measured using three biological replicates.

### 4.4. Analysis of Root Morphology

The analysis of root morphology was performed on the WINRHIZO program equipped with a Wanshen LA-S scanner (Hangzhou, China). At day 0, 3, 6, 9, and 12 after treatment, the roots of each plant under different root-zone CO_2_ treatments were separated from the shoots, and placed in a tray of water to facilitate root spreading and to keep them moist. The roots were then scanned using the above software to determine the total root length, root tip number, root surface area, and root diameter. The values were analyzed using three biological replicates.

### 4.5. Observation of Root Tip Cell Ultrastructure

Root tip samples (2–3 mm) from different root-zone CO_2_ treatments were incubated in 2.5% glutaraldehyde at 4 °C for 3 h, washed with 0.1 mol·L^−1^ phosphate buffer three times for 15 min each, and fixed with 1% osmium tetroxide at 4 °C for 2 h. The samples were washed again with a 0.1 mol·L^−1^ phosphate buffer three times for 15 min each. Graded ethanol and acetone solutions were used for dehydration. First, the samples were treated with 30%, 50%, and 70% ethanol for 15 min each; followed by treatment with 80% and 90% acetone for 15 min each; and a final treatment with 100% acetone (three times for 15 min each). The samples were then infiltrated with acetone and resin mixtures in ratios of 3:1, 1:1, and 1:3. Thereafter, the samples were embedded in resin. After polymerization (for 24 h at 37, 45, and 60 °C, respectively), the embedded tissues were sectioned using an LKB-2088 ultramicrotome (LKB-Produkter, Bramma, Sweden). The tissues were stained using uranyl acetate and lead citrate, followed by Reynold’s lead citrate. Finally, a JEX-100CX II transmission electron microscope (JEOL Ltd., Tokyo, Japan) was used to observe and image the stained sections. The observed structure included the cytoplasm, nucleus, nucleolus, plastid, mitochondria, endoplasmic reticulum, Golgi apparatus, starch granules, vacuoles, and other organelles of the middle column cells in the root tip. All observed indicators were recorded using more than 10 experimental replicates.

### 4.6. RNA Isolation, cDNA Library Construction, and Illumina Sequencing for Transcriptome Analysis

Two cm long root tips at nine days after treatment were sampled for RNA-sequencing (RNA-seq) analysis with three biological replications of each treatment (nine samples in total). To simplify the description, the three replicates of root tips at 0.037%, 0.5%, and 1.0% CO_2_ concentration treatments were designated as CK-1, -2, and -3; T1-1, -2, and -3, and T2-1, -2, and -3, respectively.

Total RNA was extracted and purified for Illumina sequencing (NEB, Ipswich, MA, USA) from the nine samples according to the NEBNext Ultra^ТМ^ RNA library prep kit. The RNA concentration was measured using a NanoDrop 2000 instrument (Thermo Fisher Scientific, Waltham, MA, USA). RNA integrity was checked on an Agilent Bioanalyzer 2100 system (Agilent Technologies, Santa Clara, CA, USA) using an RNA Nano 6000 assay kit (Agilent Technologies, Santa Clara, CA, USA). The cDNA library was then prepared and sequenced using Illumina library preparation, clustering, and sequencing according to the manufacturer’s instructions (Illumina, San Diego, CA, USA. The mRNA was purified and then fragmented, followed by cDNA synthesis. The first strand cDNA was synthesized using M-MuLV reverse transcriptase with random hexamer primers, followed by second strand synthesis using DNA polymerase I and RNase H. Using the purified cDNA as a template, and Universal and Index (X) as primers, PCR amplification of the cDNA was conducted using Phusion High-Fidelity DNA polymerase. After purification of the PCR product on an AMPure XP system, the quality of the cDNA library was verified using the Agilent Bioanalyzer 2100 system. After the cDNAs were quantified, they were clustered by Illumina’s automatic clone cluster generator, cBot. After clustering, the samples were loaded onto an Illumina instrument for automatic sequencing to generate paired end reads.

The raw reads were filtered through the platform to obtain clean reads, and were saved as FASTQ files. The final clean reads were compared with the melon reference genome sequence (http://cucurbitgenomics.org/organism/18, version 3.6.1) using the splice-aware aligner HISAT2 (version v2.1.0, http://ccb.jhu.edu/software/hisat2/index.shtml). The transcription results were assembled and summarized using String Tie (version v1.3.3, https://ccb.jhu.edu/software/stringtie/index.shtml) and Gffcompare (https://ccb.jhu.edu/software/stringtie/gffcompare.shtml), respectively. FPKM (fragment per kilogram of base fragment per million fragments) was used as an indicator to measure gene expression levels.

### 4.7. Identification of Differentially Expressed Genes (DEGs) and Functional Annotations

The DEGs were identified using DESeq, and the *p*-values were adjusted using the Benjamini and Hochberg methods to measure the false discovery rate (FDR). Among the results of the DESeq test, genes adjusted to the conditions of FDR < 0.05 and |log_2_Fold Change| ≥ 1 were defined as DEGs. Functional analysis of the DEGs was performed using GO (gene ontology) and KEGG (Kyoto Encyclopedia of Genes and Genomes) enrichment pathway analysis. In the GO-enrichment analysis, all the DEGs were mapped to the functional classification of the GO database (http://www.geneontology.org/). Thus, the gene number and GO function of every gene were obtained. Combined with the hypergeometric algorithm analysis and comparison, the significantly enriched GO functions were identified based on the entire background of all genes. For the pathway enrichment analysis, all DEGs were mapped to the KEGG database (http://www.genome.jp/kegg), and then the number of genes in every pathway was calculated. The enrichment ratios of DEGs in the KEGG pathways were counted using the KOBAS [47] software.

### 4.8. Determinations of NO_3_^−^, NH_4_^+^, and Total N Concentrations

A flow injection analyzer (Model Quik Chem 8000, Hach Co., Loveland, CO, USA) was used to determine the content of NO_3_^−^ according to Tan’s method [48]. The root concentration of NH_4_^+^ was determined according to the Berthelot reaction [49]. Root total N was determined using the combustion method with a CHN-1000 elemental analyzer (LECO Corporation, St. Joseph, MI, USA) [24]. All measured indicators were recorded using three biological replicates.

### 4.9. Determination of Nitrate Reductase (NR), Glutamine Synthetase (GS), Glutamate Synthase (GOGAT), Glutamate Dehydrogenase (GDH), Glutamic Acid Oxaloacetate Transaminase (GOT), and Glutamic-Pyruvic Transaminase (GPT) Activities

NR activity was determined according to the method of Kaiser and Huber [50]. The GS activity was determined spectrophotometrically according to Wang’s method [51]. The activities of GOGAT and GDH in roots were determined according to the method of Lin and Kao [52]. Kaur’s method was used to determine the GOT and GPT activities [53]. All measured indicators were recorded using three biological replicates.

### 4.10. Validation of Gene Expression Using qPCR

Four differentially expressed genes potentially involved in nitrogen metabolism were revealed during the transcriptomic analysis and were selected to confirm the sequencing accuracy using qPCR. The gene primers were designed using the Primer Premier software (v 6.0; PREMIER Biosoft International, Palo Alto CA, USA). The primer sequences are shown in Table 4. The cDNA was obtained using a qPCR reverse transcription kit Trans Script II (All-in-One First-Strand cDNA Synthesis Super Mix). The cDNA was used as a template and amplified using the Trans Start Top Green qPCR Super Mix of the Bio-Rad CFX96 (Bio-Rad, Hercules, CA, USA). The amplification cycling program was as follows: 94 °C for 2 min, followed by 45 cycles of 94 °C for 5 s, 60 °C for 15 s, and 72 °C for 10 s. The amount of product expression was calculated using the 2^−dCT^ method [54]. A *p*-value < 0.05 was used as the criterion for significance analysis.

### 4.11. Statistical Analysis

Data are presented as mean ± standard error (SE) and the significance of the data was analyzed using analysis of variance (ANOVA) in the SPSS 17.0 software (IBM Corp., Armonk, NY, USA). Significance analysis was performed by Duncan’s multiple range tests under conditions of *p* < 0.05. Finally, the figures were produced using Origin 8.0 (OriginLab Corporation, Northampton, MA, USA).

## 5. Conclusions

In the present study, a self-designed aeroponic culture system was employed that automatically controlled the root-zone CO_2_ concentration. The results showed that, short-term (6 day) root-zone CO_2_ treatment at 0.5% and 1.0% of oriental melon seedling roots not only increased root growth (Table 1; Table 2), but also conferred on them an improved ability to absorb NO_3_^−^-N (Figure 7) by increasing the temporary adaptive response of plants to elevated root-zone CO_2_. With the extension of treatment time, the resistance against elevated root-zone CO_2_ decreased at ninth day probably as a result of alterations to the organelle structure of the root tip cell, and the inhibition of nitrogen metabolism, as determined from the transcriptional levels, enzyme activities, and metabolites. These findings may be useful to predict how plant roots respond and adapt to elevated root-zone CO_2_ for long and short periods, and might provide key candidate genes, especially those associated with nitrogen metabolism, for the genetic improvement of oriental melons.

## Figures and Tables

**Table 1:** Root morphology index of oriental melon under elevated root-zone CO_2_.

Root Morphology Index	Days of Treatment (day)	Treatment		
		CK	T1	T2
The main root length (cm)	0	30.00 ± 3.47 a	30.00 ± 3.47 a	30.00 ± 3.47 a
	3	40.33 ± 2.09 b	52.10 ± 2.17 a	54.67 ± 2.27 a
	6	65.25 ± 4.33 a	61.07 ± 2.31 a	59.67 ± 2.23 a
	9	90.83 ± 1.19 a	83.17 ± 1.44 b	71.85 ± 1.67 c
	12	117.23 ± 2.21 a	103.8 ± 0.92 b	89.17 ± 0.98 c
Total root length (cm)	0	72.53 ± 5.52 a	72.53 ± 5.52 a	72.53 ± 5.52 a
	3	138.23 ± 6.52 b	153.19 ± 5.34 a	165.85 ± 5.25 a
	6	228.64 ± 6.26 a	233.06 ± 5.85 a	186.56 ± 5.35 b
	9	257.07 ± 5.28 a	245.21 ± 6.13 b	207.31 ± 5.98 c
	12	321.61 ± 5.69 a	299.65 ± 6.61 b	269.09 ± 5.27 c
Root surface area (cm^2^)	0	25.35 ± 0.12 a	25.35 ± 0.12 a	25.35 ± 0.12 a
	3	30.36 ± 2.45 b	35.63 ± 2.20 a	37.94 ± 3.30 a
	6	51.46 ± 6.96 a	56.00 ± 6.70 a	50.44 ± 4.63 a
	9	128.75 ± 7.33 a	102.55 ± 7.01 b	61.74 ± 5.18 c
	12	153.61 ± 7.83 a	123.69 ± 7.43 b	80.98 ± 5.54 c
Total root tip numbers	0	81.00 ± 2.21 a	81.00 ± 2.21 a	81.00 ± 2.21 a
	3	125.50 ± 5.30 b	156.80 ± 8.93 a	152.70 ± 11.80 a
	6	202.00 ± 7.86 c	233.00 ± 10.08 b	236.00 ± 8.62 a
	9	254.00 ± 6.36 a	245.00 ± 9.86 b	243.00 ± 8.09 b
	12	330.00 ± 15.72 a	247.00 ± 11.28 b	212.00 ± 9.05 c
Number of root tip 0–0.5 mm in diameter	0	22.83 ± 2.99 a	22.83 ± 2.99 a	22.83 ± 2.99 a
	3	34.23 ± 2.99 b	36.52 ± 3.39 b	45.99 ± 2.75 a
	6	86.77 ± 2.22 a	72.65 ± 2.46 b	51.37 ± 2.13 c
	9	98.49 ± 3.10 a	88.93 ± 5.17 b	77.01 ± 2.45 b
	12	118.95 ± 5.70 a	98.58 ± 5.10 b	85.68 ± 2.60 c
Number of root tip 0.5–2 mm in diameter	0	38.29 ± 3.85 a	38.29 ± 3.85 a	38.29 ± 3.85 a
	3	65.30 ± 3.99 a	45.89 ± 3.85 b	47.82 ± 4.73 b
	6	115.99 ± 5.37 a	105.18 ± 3.33 a	102.78 ± 2.54 a
	9	125.87 ± 4.10 b	122.58 ± 5.17 b	146.29 ± 4.12 a
	12	150.17 ± 4.70 b	168.22 ± 4.10 b	210.88 ± 4.60 a

Duncan’s multiple range tests were conducted: Values followed by a different small letter are significantly different at the 0.05 probability level.

**Table 2:** Plant growth index of oriental melon under elevated root-zone CO_2_.

Growth Index	Days of Treatment (day)	Treatment		
		CK	T1	T2
Root vigor (μg·g^−1^·h^−1^)	0	9.47 ± 0.87 a	9.47 ± 0.88 a	9.47 ± 0.89 a
	3	11.49 ± 0.70 b	13.01 ± 0.74 a	14.59 ± 0.82 a
	6	12.56 ± 0.67 b	15.52 ± 0.66 a	16.57 ± 0.57 a
	9	16.81 ± 0.69 a	16.13 ± 0.54 a	17.47 ± 0.70 a
	12	22.79 ± 0.83 a	17.85 ± 0.76 b	18.39 ± 0.59 b
Root volume (cm^3^)	0	2.00 ± 0.06 a	2.00 ± 0.06 a	2.00 ± 0.06 a
	3	3.33 ± 0.38 b	4.00 ± 0.38 a	5.67 ± 0.01 a
	6	9.67 ± 1.73 b	10.00 ± 0.01 a	8.67 ± 0.02 c
	9	15.67 ± 1.15 a	13.00 ± 1.01 b	11.67 ± 1.05 b
	12	36.67 ± 1.67 a	33.33 ± 1.72 b	28.33 ± 0.96 c
Shoot dry weight (g)	0	0.52 ± 0.07 a	0.52 ± 0.07 a	0.52 ± 0.07 a
	3	0.71 ± 0.00 a	0.67 ± 0.00 a	0.58 ± 0.07 b
	6	1.43 ± 0.08 a	1.38 ± 0.01 a	1.11 ± 0.09 b
	9	2.20 ± 0.06 a	1.97 ± 0.25 a	1.53 ± 0.16 b
	12	4.30 ± 0.26 a	3.20 ± 0.39 b	2.27 ± 0.21 c
Root dry weight (g)	0	0.12 ± 0.03 a	0.12 ± 0.03 a	0.12 ± 0.03 a
	3	0.21 ± 0.00 a	0.20 ± 0.00 a	0.21 ± 0.02 a
	6	0.36 ± 0.04 b	0.42 ± 0.04 a	0.39 ± 0.08 a
	9	0.60 ± 0.05 a	0.41 ± 0.10 b	0.33 ± 0.07 c
	12	1.07 ± 0.07 a	0.82 ± 0.08 b	0.54 ± 0.08 c

Duncan’s multiple range tests were conducted: Values followed by a different small letter are significantly different at the 0.05 probability level.

**Table 3:** Differentially expressed genes (DEGs) annotated in nitrogen metabolism in T1/CK and T2/CK comparisons. Signification of DEGs was measured by the two criterions of |Log_2_FoldChange| ≥ 1 and FDR ≤ 0.05.

Different Expressed Genes		T1/CK		
Gene ID	Gene Name	FDR	Log_2_FoldChange	Regulation
MELO3C000140.2	High-affinity nitrate transporter 2.2	0.007581	1.219896	Up
MELO3C010444.2	Nitrate reductase	5.21 ± 10^−9^	1.085544	Up
MELO3C009476.2	Alpha carbonic anhydrase	0.030669	1.104078	Up
**Different Expressed Genes**		**T2/CK**		
**Gene ID**	**Gene Name**	**FDR**	**Log_2_FoldChange**	**Regulation**
MELO3C010444.2	Nitrate reductase	4.40 ± 10^−31^	1.903265	Up
MELO3C009476.2	Alpha carbonic anhydrase	0.009895	1.286820	Up
MELO3C024476.2	Alpha carbonic anhydrase 7-like	9.99 ± 10^−5^	−1.256327	Down

FDR: Statistical difference significance value. Log_2_FoldChange: Difference significance determined by the threshold value. Regulation: Gene relative expression status compared to CK.

**Table 4:** QPCR primer sequences used to validate the RNA-seq data.

Gene ID		Sequence (5′-3′)
actin	F	AAGGCAAACAGGGAGAAGATGA
	R	AGCAAGGTCGAGACGTAGGATA
MELO3C000140.2	F	GATGGCGAATCTAGTGGCTAG
	R	GTCCACAACCTTCCTCTCATC
MELO3C010444.2	F	TGGTGTATGCGAATAGAACGG
	R	CACACTATATTCCCACCCTTCC
MELO3C009476.2	F	CTTCATCTAGTCCATCAGGCAG
	R	GTGCTTTGTGTCTAGGTCTCC
MELO3C024476.2	F	GAAGATAAGGACAGTGTCAAGG
	R	GAGTTGGCCGATAGAGACCG

F: Forward. R: Reverse.

## Supplementary Materials

Supplementary materials can be found at https://www.mdpi.com/1422-0067/21/3/803/s1.
